# Analysis of detoxification kinetics and end products of furan aldehydes in *Acinetobacter baylyi* ADP1

**DOI:** 10.1038/s41598-024-81124-4

**Published:** 2024-11-29

**Authors:** Changshuo Liu, Elena Efimova, Ville Santala, Suvi Santala

**Affiliations:** Faculty of Engineering and Natural Sciences, Tampere University, Hervanta Campus, PO Box 527, Tampere, FI-33014 Finland

**Keywords:** *Acinetobacter baylyi* ADP1, Lignocellulosic hydrolysate, Furan aldehydes, Furfural, 5-Hydroxymethylfurfural, Biodetoxification, Biotechnology, Industrial microbiology

## Abstract

The efficient utilization of lignocellulosic hydrolysates in bioprocesses is impeded by their complex composition and the presence of toxic compounds, such as furan aldehydes, formed during lignocellulose pretreatment. Biological detoxification of these furan aldehydes offers a promising solution to enhance the utilization of lignocellulosic hydrolysates. *Acinetobacter baylyi* ADP1 is known to metabolize furan aldehydes, yet the complete spectrum of reaction products and dynamics remains unclear. Here, we determined the detoxification metabolites of furfural and 5-hydroxymethylfurfural in *A. baylyi* ADP1 and studied the kinetics of detoxification. The results indicate that detoxification in *A. baylyi* ADP1 follows a typical alcohol-aldehyde-acid scheme, with furoic acid and 5-hydroxymethyl-2-furancarboxylic acid as the final products for furfural and 5-hydroxymethylfurfural, respectively. Both end products were found to be less toxic for cells than their unmodified forms. These findings underscore the potential of *A. baylyi* ADP1 in detoxifying lignocellulosic hydrolysates for bioprocess applications.

**Supplementary Information:**

The online version contains supplementary material available at 10.1038/s41598-024-81124-4.

## Introduction

Lignocellulosic biomass, composed of cellulose, hemicellulose, and lignin, is a renewable and abundant resource for producing e.g., biofuels, value-added chemicals, and bioplastics by microorganisms^1,2^. However, its efficient utilization is challenged by several factors, including the presence of inhibitory compounds like furan aldehydes, which form during lignocellulose pretreatment^3,4^. Furan aldehydes, including furfural and 5-hydroxymethylfurfural (HMF), exert toxic effects on microbial cells through various mechanisms, including the inhibition of metabolic pathways, DNA damage, and redox stress^5^, hindering the cell growth and bioproduction^6,7^.

Many microorganisms possess various mechanisms to detoxify and convert these furan aldehydes into less harmful derivatives. For example, *Saccharomyces cerevisiae* and *Escherichia coli* convert furan aldehydes to their corresponding alcohols: furfural is converted to furfuryl alcohol (FOH), and HMF is converted to 2,5-bis(hydroxymethyl)furan (BHMF)^8–10^. In order to enhance the utilization of lignocellulosic biomass, some microbes, such as *Fusarium striatum*^11^, have been employed as biological detoxifiers for lignocellulosic hydrolysates.

*Acinetobacter baylyi* ADP1 (later ADP1) is a naturally competent soil bacterium with a versatile metabolism. It has been extensively used as a model system for genetic and metabolic studies^12–14^ and has been engineered for the production of various value-added compounds^15^, such as wax esters^16–18^, alkanes and 1-alkenes^18–20^, mevalonate^21^, and *cis*,* cis*-muconic acid^22^. In addition, ADP1 has been also employed for the detoxification of several inhibitors present in lignocellulosic hydrolysates, including acetic acid, aromatic compounds, and furan aldehydes^23–25^.

While the aromatic degradation pathways of ADP1 are well-characterized^26,27^, the biotransformation of furan aldehydes is less understood. Recently, the transformation of furfural by ADP1 was characterized^28^. Although ADP1 is known to convert also HMF^25^, the specific transformation product of HMF has not been identified. However, another *Acinetobacter* strain has been reported to convert HMF to 2,5-furandicarboxylic acid (FDCA)^29^.

In this study, we investigated the kinetics and determined the metabolites of furfural and HMF biotransformation in ADP1. We also evaluated the toxicity of the furan aldehydes and their metabolites for ADP1. Our findings demonstrate the potential of ADP1 as a host strain for the biological detoxification of the furan aldehydes and further elucidate the underlying mechanisms.

## Methods

### Strains

A wild-type *A. baylyi* ADP1 (DSM 24193, DSMZ, Germany) was used to investigate the biotransformation of furan aldehydes. A bioluminescence reporter strain ADP1Δ*acr1*::*kan*^*R*^ [*luxCDABE*] (later ADP1 3383 F)^30^, was used to test the toxicity of furan aldehydes and their biotransformation products.

### Media and cultivation conditions

Low-salt lysogeny broth (LB)-agar (tryptone 10 g/L, yeast extract 5 g/L, NaCl 1 g/L, 15 g/L agar) supplemented with 0.2% (w/v) glucose was used for all ADP1 inoculations. For the identification of furan aldehydes’ biotransformation products, ADP1 cells were precultivated in 25 mL mineral salts medium (MSM, composition: K_2_HPO_4_ 3.88 g/L, NaH_2_PO_4_ 1.63 g/L, (NH_4_)_2_SO_4_ 2.00 g/L, MgCl_2_ · 6H_2_O 0.1 g/L, ethylenediaminetetraacetic acid (EDTA) 10 mg/L, ZnSO_4_ · 7H_2_O 2 mg/L, CaCl_2_ · 2H_2_O 1 mg/L, FeSO_4_ · 7H_2_O 5 mg/L, Na_2_MoO_4_ · 2H_2_O 0.2 mg/L, CuSO_4_ · 5H_2_O 0.2 mg/L, CoCl_2_ · 6H_2_O 0.4 mg/L, MnCl_2_ · 2H_2_O 1 mg/L)^31^ supplemented with 50 mM acetate, 0.2% (w/v) casein amino acids, and 2 mM furfural or 2 mM HMF in 250 mL Erlenmeyer flasks, incubated at 30 °C and 300 rpm for overnight. The cells from the preculture were collected and washed using fresh MSM before inoculations. The main cultivations were carried out in 25 mL MSM supplemented with 50 mM acetate, and 10 mM furfural or 8 mM HMF in 250 mL Erlenmeyer flasks, incubated at 30 °C and 300 rpm. Samples of 1 mL were collected and centrifuged at 13,500 g for 3 min, and the supernatants were analyzed using high performance liquid chromatograph (HPLC) and liquid chromatography-mass spectrometry (LC-MS).

For the luminescence-based toxicity test, ADP1 3383F^30^ cells were precultivated in 5 mL LB medium (tryptone 10 g/L, yeast extract 5 g/L, NaCl 1 g/L) supplemented with 0.4% (w/v) glucose in 14 mL culture tubes, incubated at 30 °C and 300 rpm for overnight. The cells from preculture were collected, washed, and resuspended using fresh MSM to remove all the carbon sources. The cell resuspensions (final optical density at 600 nm 0.25) were added to the solutions (5 mM, 10 mM, and 15 mM) of furfural, FOH, furoate (the potassium salt of furoic acid), HMF, BHMF, and 5-Hydroxymethyl-2-furancarboxylate (K-HMFCA, the potassium salt of 5-hydroxymethyl-2-furancarboxylic acid) in 96-well plate (Greiner Bio-One CellStar *µ*Clear), incubated and measured the luminescence using Spark multimode microplate reader (Tecan, Switzerland) at 30 °C, with final volume of 200 µL.

### Analytical methods

HPLC analysis of furfural, FOH, furoic acid, HMF, BHMF, and 5-hydroxymethyl-2-furancarboxylic acid (HMFCA) was performed on the instrument Shimadzu LC-40D (Japan) equipped with the photo diode array detector Shimadzu SPD-M40 (Japan). The compounds were analyzed on the column Luna C18, 150 × 4.6 mm, 5 μm (Phenomenex, USA) at 40 °C. The mixture 0.1% formic acid: methanol (95:5, v/v) was used as eluent with the flow rate 1.0 mL/min.

LC-MS analysis of furan derivatives was done on Agilent 1260 Infinity chromatograph (USA) connected with JEOL AccuTOF LC Plus (JMS-T100LP) mass spectrometer (Japan). LC-MS was performed on Poroshell 120 EC-18 column, 100 × 4.6 mm, 2.7 µ (Agilent, USA) at 30 °C using elution with 0.1% formic acid: methanol (95:5, v/v), flow 0.2 mL/min.

Experiments were conducted in independent biological triplicates. Mean values and standard deviations were calculated using Microsoft Excel, with the AVERAGE and STDEV functions applied to each set of measurements.

## Results

### Identification of the biotransformation products of furfural and HMF

**Fig. 1:**
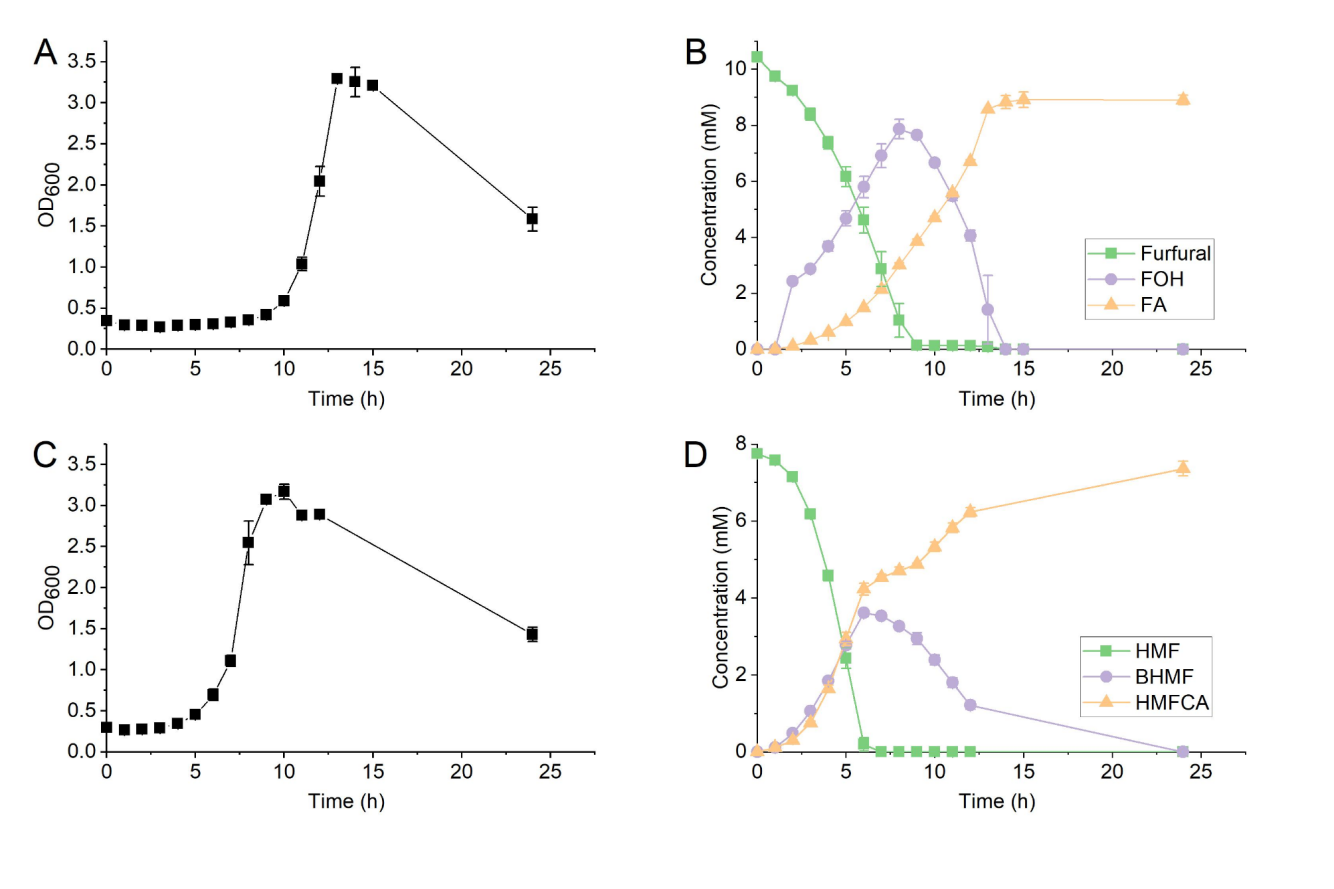
Bioconversion kinetics of furfural and HMF in ADP1. Cell growth profile (**A**), and consumption and production (**B**) of furfural and its metabolites; cell growth profile (**C**), and consumption and production (**D**) of HMF and its metabolites. The concentration of HMF at the 6–12 h is marked as 0, although traces of HMF (concentration below 0.1 mM) was detected by HPLC, but the concentration cannot be reliably calculated. FOH, furfuryl alcohol; FA, furoic acid; HMF, 5-hydroxymethylfurfural; BHMF, 2,5-bis(hydroxymethyl)furan; HMFCA, 5-hydroxymethyl-2-furancarboxylic acid. The experiment was repeated using independent biological triplicates. The averages of the measurements, with error bars representing standard deviations, are shown.

Batch cultivations were carried out to investigate the biotransformation of the furan aldehydes in ADP1. Furfural and HMF were individually added to the medium at concentrations 10 mM and 8 mM, respectively. Acetate was added to the medium as the carbon source because acetate is a typical product from the lignocellulosic depolymerizations^3^ and a preferred carbon source of ADP1^23,25^. As a result, similar amounts of biomass were accumulated in both cultures (Fig. 1A,C) after 24 h cultivations.

The samples of the cultivations were first analyzed using HPLC. Consumption of both furfural and HMF was clearly observed. In the furfural cultivations, two signals were detected with maximum absorbance wavelengths (A_max_) at 220 nm (metabolite #1) and 254 nm (metabolite #2). Similarly, in the HMF cultivations, two major metabolites were detected with A_max_ at 220 nm (metabolite #3) and 254 nm (metabolite #4).

To identify the compounds corresponding to the detected signals, the samples were further analyzed using LC-MS. In the furfural cultivations, metabolite #1 was not clearly identified by LC-MS, while metabolite #2 showed a molecular ion [M-H]^−^ 110.99771 corresponding to isotope model of furoic acid (*m/z* calculated for C_5_H_3_O_3_^−^ [M-H]^−^ 111.00877), A_max_ 254 nm. In the HMF cultivations, the metabolites #3 and #4 exhibited molecular ions [M + H]^+^ 129.10341 and [M-H]^−^ 141.00761, corresponding to isotope models of 2,5-bis(hydroxymethyl)furan (BHMF, *m/z* calculated for C_6_H_9_O_3_^+^ [M + H]^+^ 129.05462), A_max_ 220 nm, and 5-hydroxymethyl-2-furancarboxylic acid (HMFCA, *m/z* calculated for C_6_H_5_O_4_^−^ [M-H]^−^ 141.01933), A_max_ 254 nm, respectively. The metabolites were further confirmed using standards of furfuryl alcohol (FOH), furoic acid, BHMF, and HMFCA in LC-MS and HPLC (see Supplementary Figure S1 online). Thus, the metabolites from furfural conversion were identified as furfuryl alcohol and furoic acid, while those from HMF were identified as BHMF and HMFCA (Table 1).

After identifying the detected compounds, the conversion kinetics of furfural, HMF, and their metabolites were determined (Fig. 1B,D). In the batch cultivation, furfural was completely consumed after 14 h. FOH was detected in the samples from the 2 h to 14 h, with the concentration first increasing and then decreasing, reaching its maximum concentration at the 8 h timepoint. Furoic acid was detected already in the 1 h sample. After 14 h, only furoic acid was detected, suggesting it to be the end-product of the furfural detoxification. The yield of furoic acid at 24 h was 0.85 ± 0.02 g/g _furfural_ (mean ± standard deviation).

HMF and its metabolites exhibited a similar trend as that of furfural (Fig. 1D). HMF was almost completely consumed within 12 h. BHMF and HMFCA began to accumulate from the start until the 6 h timepoint. After this point, the concentration of BMHF in the samples started to decrease, while the concentration of HMFCA continued to increase. BMHF was totally depleted by 24 h. Thus, the main end-product from the detoxification of HMF was HMFCA. The yield of HMFCA at 24 h was 0.95 ± 0.02 g/g _HMF_ (mean ± standard deviation).

We also detected another metabolite in the HMF cultivations, with a molecular ion [M-H]^−^ 138.99137 and A_max_ corresponding to isotope model of 5-formylfuran-2-carboxylic acid (FFCA, *m/z* calculated for C_6_H_3_O_4_^−^ [M-H]^−^ 139.00368), A_max_ 280 nm. This metabolite constituted a minor proportion, less than 5%, with a concentration below 0.4 mM, as calculated by the yield of the main end-product, HMFCA. Additionally, the dynamics of the formation of this compound was not observed to be directly related to BHMF and HMFCA.

**Table 1:** Identified major metabolites of furfural and HMF in ADP1.

Metabolite number	Metabolites	Molecular ion	A_max_ (nm)
Furfural as substrate			
#1	Furfuryl alcohol (FOH)	-	220
#2	Furoic acid	[M-H]^−^ 110.99771	254
HMF as substrate			
#3	2,5-Bis(hydroxymethyl)furan (BHMF)	[M + H]^+^ 129.10341	220
#4	5-Hydroxymethyl-2-furancarboxylic acid (HMFCA)	[M-H]^−^ 141.00761	254

The molecular ion was based on LC-MS analysis, while A_max_ was based on HPLC-PDA analysis.

**Fig. 2:**
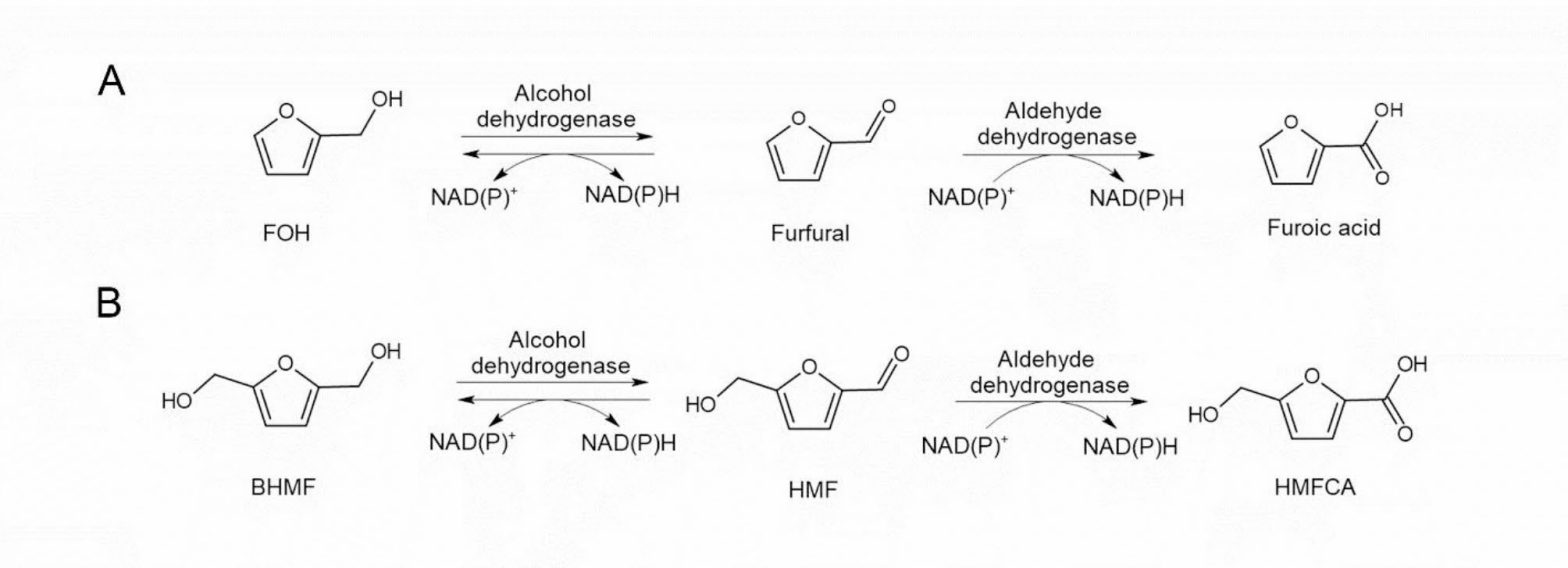
Proposed metabolic detoxification schemes of furfural (**A**) and HMF (**B**) in ADP1. FOH, furfuryl alcohol; HMF, 5-hydroxymethylfurfural; BHMF, 2,5-bis(hydroxymethyl)furan; HMFCA, 5-hydroxymethyl-2-furancarboxylic acid.

According to the kinetics of the furan aldehydes and their metabolites observed in this study, it is likely that the metabolic pathway of the furan aldehydes in ADP1 follows a typical alcohol-aldehyde-acid pathway, facilitated by alcohol dehydrogenase and aldehyde dehydrogenase enzymes (Fig. 2).

### Toxicity assay of furan aldehydes and their metabolites

**Fig. 3:**
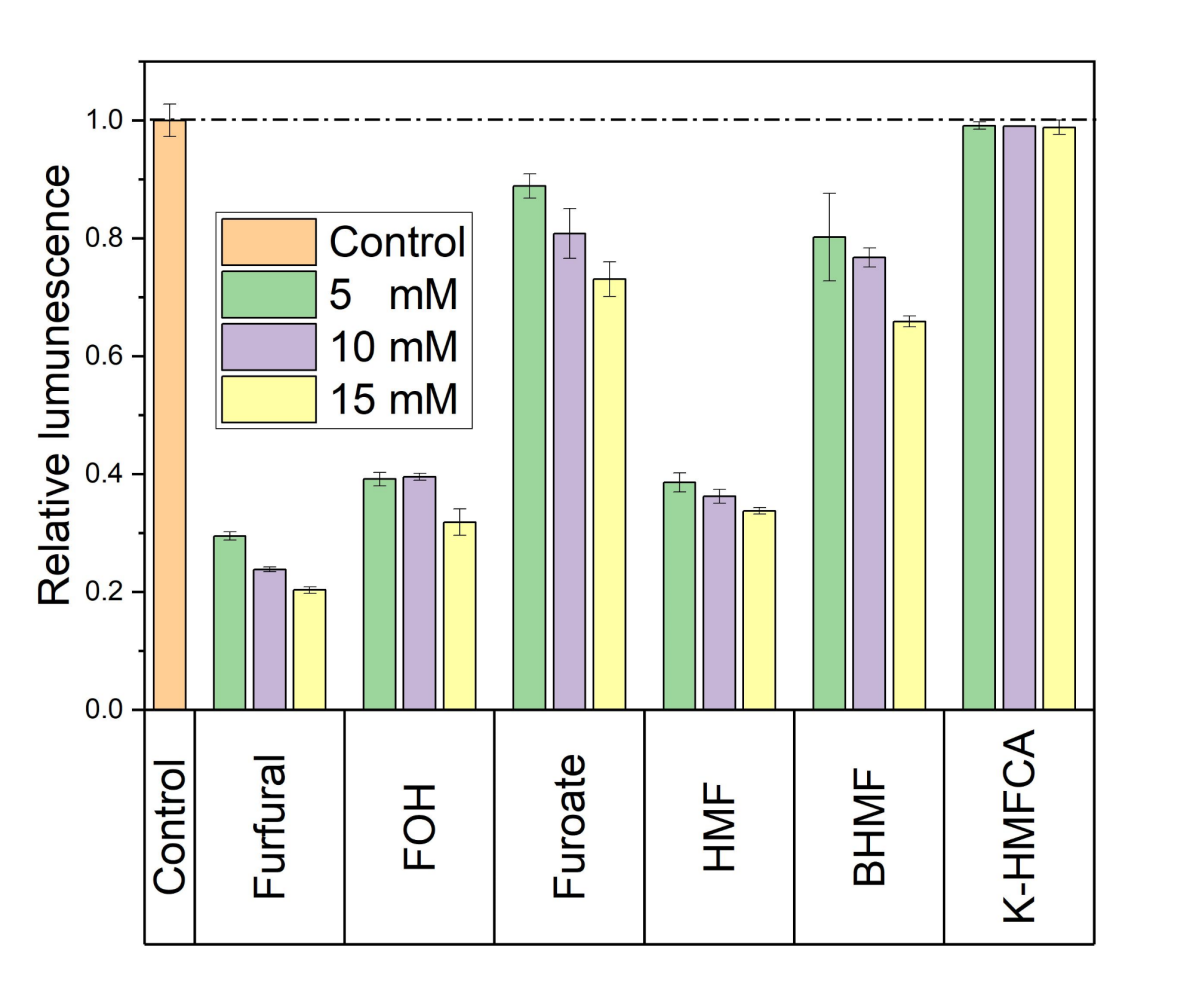
Relative luminescence of cells exposed to the furan derivatives. Exposure time was 5 min. FOH, furfuryl alcohol; HMF, 5-hydroxymethylfurfural; BHMF, 2,5-bis(hydroxymethyl)furan; K-HMFCA, 5-hydroxymethyl-2-furancarboxylic acid potassium salt. The luminescence signal of the control was set as relative luminescence of 1. The experiment was repeated using independent biological triplicates. The averages of the measurements, with error bars representing standard deviations, are shown.

To investigate the toxicity of these furan compounds and their derivatives to ADP1 cells, a bioluminescent ADP1 strain 3383F^30^ was used for the toxicity test. The cells of 3383F were exposed to the furan derivatives at different concentrations and luminescence was measured after 5 min incubation (Fig. 3). For the control, only the medium was used. To eliminate any potential effects caused by the acidity of the compounds, we used the potassium salt of 5-hydroxymethyl-2-furancarboxylic acid (K-HMFCA) and the potassium salt of furoic acid (furoate). The cells showed the lowest luminescence in the presence of furfural, indicating it has the highest toxicity effect: the luminescence was 70% lower compared to that of the control.

For furfural and its metabolites, the highest luminescence signal and therefore the lowest toxicity effect was observed when the cells were exposed to furoate; and exposure to furfural resulted in the lowest luminescence suggesting the highest toxicity effect. The same trend was observed also with HMF and its metabolites. In addition, except for K-HMFCA, the luminescence decreased along with the increasing concentrations of the compounds. When the cells were exposed to K-HMFCA at various concentrations, the luminescence was indistinguishable from the control.

Furthermore, a growth-based test was conducted using wild-type ADP1 to confirm the effect of furoate and K-HMFCA on growth (see Supplementary Figure S2 online). Interestingly, the growth curves of the cells in all tested concentrations of furoate or K-HMFCA were similar, indicating that the cell growth was not affected by these compounds. However, using the luminescence-based assay, we observed that furoate had a slight effect on the cells, observed as decreased luminescence signal. This can be attributed to the high sensitivity of the luminescence assay compared to the growth-based test.

## Discussion

Furan aldehydes, particularly furfural and HMF, pose significant concerns in lignocellulose-based biomanufacturing due to their toxicity to host strains^4,32^. While various methods, such as co-treatment, have been developed to reduce the recalcitrance and toxicity of lignocellulosic hydrolysates^33^, it is crucial to consider the impact of furan aldehyde formation, especially with commonly used pretreatment methods, such as acidic thermal treatment^34^, when developing strategies for efficient lignocellulosic biomass utilization.

However, many microorganisms can naturally detoxify these furan aldehydes through oxidation or reduction reactions, typically resulting in the corresponding alcohol or acid forms as end-metabolites^35^. As an example of such a microorganism, ADP1 represents a promising candidate for the biological detoxification of the furan aldehydes in lignocellulosic hydrolysates^23,25^.

In this study, we investigated the conversion of furfural and HMF in ADP1 using LC-MS and HPLC to identify the metabolites. For the cultivations, we used 10 mM of furfural and 8 mM of HMF, which equate to approximately 1 g/L, to ensure a high-resolution instrument analysis. These concentrations are comparable to or higher than those previously found for example in a rice straw hydrolysate^36^ and spruce-based hydrolysates^37,38^.

Based on the analyses, furfural and HMF can be converted into FOH and furoic acid, and BHMF and HMFCA, respectively, with the final end-products for each furan aldehyde being the acid form. These end-products have also been reported in other microorganisms such as *Acetobacter rancens*, *Serratia liquefaciens*,* and Ureibacillus thermosphaericus*, as reviewed by Yuan et al.^35^. The additional compound detected, possibly FFCA, is likely associated with reactions by nonspecific oxidoreductases, which have been reported to oxidize HMFCA to FFCA in other strains^35,39^.

In the previous study, difurfuryl-ether was reported as the end product of furfural conversion in ADP1^28^. However, we did not observe difurfuryl-ether in this study. These differences can potentially be attributed to the different analytical methods used; in the previous study, a liquid-liquid continuous extraction method was applied to purify the difurfuryl-ether^28^. In our study, by contrast, we directly analyzed the supernatant from the cultivations. Based on our data, the final yield of furoic acid and HMFCA were approximately 85% and 95%, respectively. The remaining fractions of furfural and HMF, which were not accounted for, may have been converted into other compounds that were undetectable under the analytical conditions used.

Based on the results, we suggest the detoxification route in ADP1 to occur via a typical alcohol-aldehyde-acid pathway (Fig. 2). This is also supported by the study of Arteaga et al., who employed transcriptional analyses to identify several genes, such as aryl-alcohol dehydrogenase (*areB*, ACIAD1429) and alcohol dehydrogenase (*frmA*, ACIAD1879), that were overexpressed when ADP1 was exposed to furfural^28^. In *P. putida* KT2440, the HMF conversion was greatly reduced by deleting genes encoding an enzyme complex PaoEFG and aldehyde dehydrogenases AldB-I, II^39^. Interestingly, ADP1 possesses a homologous acetaldehyde dehydrogenase (*acoD*, ACIAD2018) to AldB-I and AldB-II of *P. putida* KT2440, with the amino acid sequence of AcoD exhibiting a sequence identity of 68.38% to AldB-I and 67% to AldB-II. Thus, these ADP1 genes represent logical candidates for genes encoding activities related to the detoxification of HMF and furfural in future studies. However, due to the large number of the potential gene candidates related to furan aldehyde biotransformation in ADP1, verification of the genetic basis of the pathways will remain as a future investigation.

The growth-inhibiting effects of furfural and HMF were evident, as cell growth only initiated after most of the added furfural and HMF were consumed in both batch cultivations (Fig. 1). To further investigate the relative toxicity effects of the different furan aldehydes and their derivatives, we employed a bioluminescence-based assay. The luminescence production by bacterial luciferase is linked to several essential cellular factors, such as cofactors and ATP availability, thus rapidly and sensitively reflecting the changes in the viability and metabolic state of the cells^40^. The assay demonstrated that the products resulting from ADP1 transformations were less toxic than the original compounds furfural and HMF.

Furthermore, it was found that furfural is more toxic than HMF to ADP1. These results align with previous reports for *E. coli*^41,42^. The furan acid forms exhibited the lowest toxicity to ADP1 compared to their corresponding alcohol and aldehyde forms. Of note, industrially well-established workhorse strains, such as *E. coli* and *S. cerevisiae*, primarily transform furfural and HMF to their alcohol forms^8–10^, which in our study were found to be more toxic for ADP1 than the end-products produced by ADP1. Thus, the further conversion of FOH and BHMF to their acid forms by ADP1 would likely decrease the toxicity also for these widely used workhorses, which supports for example the use of synthetic consortia involving ADP1, as proposed previously^25^.

The relative toxicity of these furan derivatives rationalizes the observed biotransformation process: initially, ADP1 simultaneously converts the furan aldehydes into their alcohol and acid forms hypothetically by nonspecific dehydrogenases, rapidly removing the most toxic compounds, furan aldehydes. When most of the furan aldehydes are depleted, the cells convert the furan alcohols back into the furan aldehydes and then rapidly into the final end-metabolite with the lowest toxicity, furan acids. During the transformation process from furan alcohols to furan acids, the concentration of the furan aldehydes was detected at trace levels, supporting this mechanism (Fig. 1A and 9–13 h; Fig. 1B and 6–12 h).

The investigation of biodetoxification of the furan aldehydes in ADP1 further confirms its potential as a detoxifier of lignocellulosic feedstock. Our recent work established a synthetic bacterium-yeast consortium of ADP1 and *S. cerevisiae* for the efficient valorization of lignocellulosic substrates, with ADP1 proving more efficient than *S. cerevisiae* in the bioconversion of these furan aldehydes, improving the productivity of lactic acid in *S. cerevisiae*^25^. Clarifying the detoxification mechanism paves the way for further metabolic studies in ADP1 to improve the valorization and carbon recovery of lignocellulose^32^; for example, ADP1 could be employed for the utilization of the furan derivatives as carbon sources by applying the *hmf*-encoding gene clusters identified previously^43,44^ or valorization of HMF to high-value products, such as 2,5-furandicarboxylic acid (FDCA)^35^. Taken together, ADP1 possesses great potential for the bioprocesses using lignocellulosic feedstock.

## Electronic supplementary material

Below is the link to the electronic supplementary material.

Supplementary Material 1

## Data Availability

Data associated with the present study will be available upon request from the corresponding author.

## Abbreviations

FOH: Furfuryl alcohol
HMF: 5-Hydroxymethylfurfural
BHMF: 2,5-Bis(hydroxymethyl)furan
HMFCA: 5-Hydroxymethyl-2-furancarboxylic acid
K-HMFCA: Potassium salt of 5-hydroxymethyl-2-furancarboxylic acid
FFCA: 5-Formylfuran-2-carboxylic acid
FDCA: 2,5-Furandicarboxylic acid
ADP1: *Acinetobacter baylyi* ADP1
MSM: Mineral salts medium
LB: Lysogeny broth
EDTA: Ethylenediaminetetraacetic acid
HPLC: High performance liquid chromatograph
LC-MS: Liquid chromatography-mass spectrometry
OD_600_: Optical density at 600 nm
