# Supplementary material for: Analysis of detoxification kinetics and end products of furan aldehydes in Acinetobacter baylyi ADP1
Source: Sci Rep. 2024 Nov 29;14:29678. doi: 10.1038/s41598-024-81124-4 (PMC11607399; doi:10.1038/s41598-024-81124-4)
Supplement: Supplementary file 1 — Supplementary Material 1 [file 41598_2024_81124_MOESM1_ESM.docx]

**Supplementary information**

**Analysis of detoxification kinetics and end products of furan aldehydes in *Acinetobacter baylyi* ADP1**

Changshuo Liu^1^, Elena Efimova^1^, Ville Santala^1^, and Suvi Santala^1*^

^1^ Faculty of Engineering and Natural Sciences, Hervanta Campus, Tampere University, PO Box 527, FI-33014 Tampere, Finland

Changshuo Liu: [changshuo.liu@tuni.fi](mailto:changshuo.liu@tuni.fi) (ORCID: 0009-0001-1544-3694)

Elena Efimova: [elena.efimova@tuni.fi](mailto:elena.efimova@tuni.fi)

Ville Santala: [ville.santala@tuni.fi](mailto:ville.santala@tuni.fi) (ORCID: 0000-0002-9084-931X)

Suvi Santala: [suvi.santala@tuni.fi](mailto:suvi.santala@tuni.fi) (ORCID: 0000-0002-0047-5319)

^*^Corresponding author: Suvi Santala (suvi.santala@tuni.fi)

**Abbreviations**

FOH Furfuryl alcohol

HMF 5-Hydroxymethylfurfural

BHMF 2,5-Bis(hydroxymethyl)furan

HMFCA 5-Hydroxymethyl-2-furancarboxylic acid

K-HMFCA Potassium salt of 5-hydroxymethyl-2-furancarboxylic acid

MSM Mineral salts medium


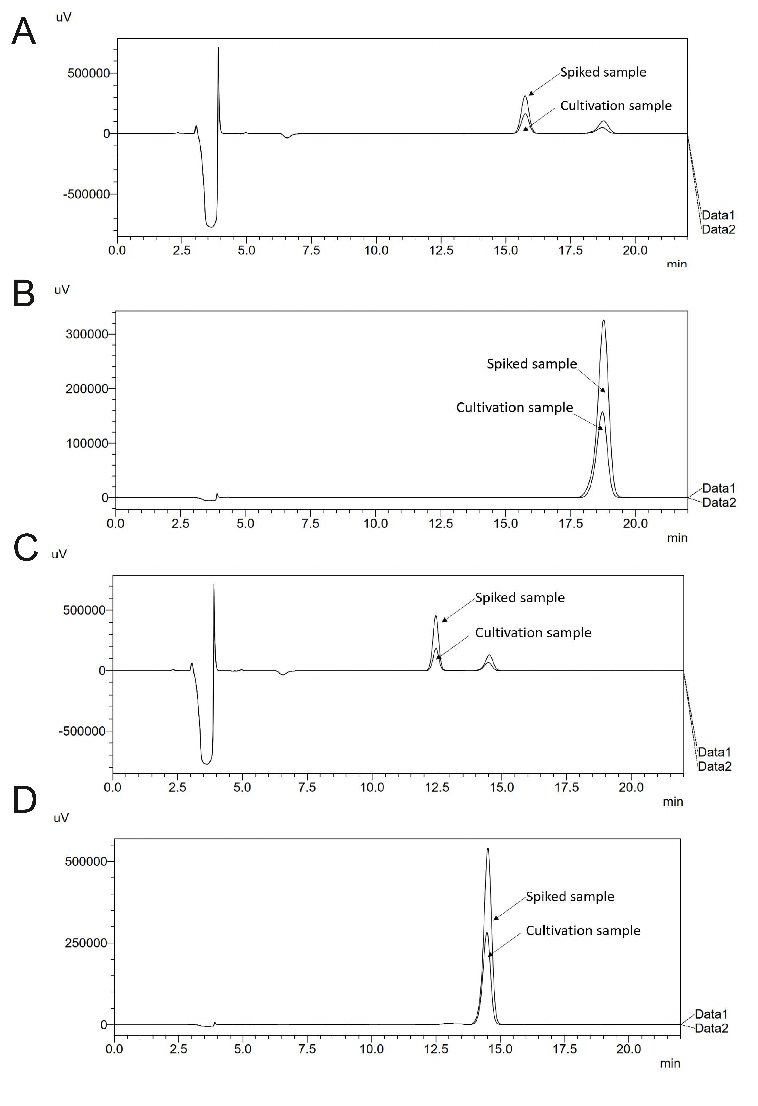


**Supplementary Figure S1**. Overlayed HPLC chromatographs of cultivation samples and samples spiked with standards. (A) 9 h sample of furfural cultivation (Data 1, Cultivation sample) and the sample spiked with 1 mM FOH (Data 2, Spiked sample), extracted at 254 nm; (B) 9 h sample of furfural cultivation (Data 1, Cultivation sample) and the sample spiked with 1 mM furoic acid (Data 2, Spiked sample), extracted at 220 nm; (C) 6 h sample of HMF cultivation (Data 1, Cultivation sample) and the sample spiked with 1 mM BHMF (Data 2, Spiked sample), extracted at 254 nm; (D) 9 h sample of HMF cultivation (Data 1, Cultivation sample) and the sample spiked with 1 mM HMFCA (Data 2, Spiked sample), extracted at 220 nm.

**

Supplementary Figure S2.** Cell growth of ADP1 in the presence of furoate (the potassium salt of furoic acid) (A) and K-HMFCA (B). Cultivations without furoate and K-HMFCA were used as controls. ADP1 cells were precultivated in 5 mL MSM supplemented with 50 mM acetate, 0.2 % (*w/v*) casein amino acids in 14 mL culture tubes, incubated at 30 °C and 300 rpm for overnight. Then the cells were inoculated to 200 µL MSM supplemented with 50 mM acetate and 5 or 10 or 15 mM furoate or HMFCA in a 96 well-plate. The plate was incubated at 30 °C, shaken regularly, and the optical density at 600 nm (OD_600_) was monitored by Spark multimode microplate reader (Tecan, Switzerland) at 30 °C. The experiment was repeated using independent biological triplicates. The averages of the measurements, with error bars representing standard deviations are shown.
